# COVID‐19 and vertical transmission: assessing the expression of ACE2/TMPRSS2 in the human fetus and placenta to assess the risk of SARS‐CoV‐2 infection

**DOI:** 10.1111/1471-0528.16974

**Published:** 2021-11-18

**Authors:** MA Beesley, JR Davidson, F Panariello, S Shibuya, D Scaglioni, BC Jones, K Maksym, O Ogunbiyi, NJ Sebire, D Cacchiarelli, AL David, P De Coppi, MFM Gerli

**Affiliations:** ^1^ Great Ormond Street Institute of Child Health University College London UK; ^2^ EGA Institute for Women’s Health University College London UK; ^3^ Telethon Institute of Genetics and Medicine (TIGEM) Armenise/Harvard Laboratory of Integrative Genomics Pozzuoli Italy; ^4^ Department of Translational Medicine University of Naples ‘Federico II’ Naples Italy; ^5^ NIHR Great Ormond Street Biomedical Research Centre London UK; ^6^ Great Ormond Street Hospital for Children London UK; ^7^ Fetal Medicine Unit University College London NHS Foundation Trust London UK; ^8^ UCL Division of Surgery and Interventional Science Royal Free Hospital London UK

**Keywords:** ACE2, COVID‐19, fetal infection, SARS‐CoV2, TMPRSS2, vertical transmission

## Abstract

**Background:**

Pregnant women have been identified as a potentially at‐risk group concerning COVID‐19 infection, but little is known regarding the susceptibility of the fetus to infection. Co‐expression of ACE2 and TMPRSS2 has been identified as a prerequisite for infection, and expression across different tissues is known to vary between children and adults. However, the expression of these proteins in the fetus is unknown.

**Methods:**

We performed a retrospective analysis of a single cell data repository. The data were then validated at both gene and protein level by performing RT‐qPCR and two‐colour immunohistochemistry on a library of second‐trimester human fetal tissues.

**Findings:**

TMPRSS2 is present at both gene and protein level in the predominantly epithelial fetal tissues analysed. ACE2 is present at significant levels only in the fetal intestine and kidney, and is not expressed in the fetal lung. The placenta also does not co‐express the two proteins across the second trimester or at term.

**Interpretation:**

This dataset indicates that the lungs are unlikely to be a viable route of SARS‐CoV2 fetal infection. The fetal kidney, despite presenting both the proteins required for the infection, is anatomically protected from the exposure to the virus. However, the gastrointestinal tract is likely to be susceptible to infection due to its high co‐expression of both proteins, as well as its exposure to potentially infected amniotic fluid.

**Tweetable abstract:**

This work provides detailed mechanistic insight into the relative protection & vulnerabilities of the fetus & placenta to SARS‐CoV‐2 infection by scRNAseq & protein expression analysis for ACE2 & TMPRSS2. The findings help to explain the low rate of vertical transmission.

## Tweetable abstract

This work provides detailed mechanistic insight into the relative protection & vulnerabilities of the fetus & placenta to SARS‐CoV‐2 infection by scRNAseq & protein expression analysis for ACE2 & TMPRSS2. The findings help to explain the low rate of vertical transmission.

## Introduction

The COVID‐19 pandemic has brought about unprecedented research efforts in attempts to prevent and treat infection across all ages. SARS‐CoV‐2 viral entry mechanisms into the human body have been well established, highlighting the importance of tissue‐level co‐expression of ACE2 and TMPRSS2 (along with potential roles for further surface markers, such as NRP1).^1^, ^2^ The risk profile and infection mechanisms of SARS‐CoV‐2 appear significantly different in young children than in older individuals. Clinical reports of confirmed perinatal infection in the neonate have led to concerns of a potential mechanism for vertical transmission. Recent data suggest that the fetus may have some susceptibility to SARS‐CoV‐2 infection,^3^, ^4^, ^5^ alongside a plausible increased risk of preterm birth. These observed risks appear to relate to the severity of maternal infection, and documented placental pathology has been reported to correlate with severe maternal symptoms.^6^, ^7^

Documented cases of confirmed vertical transmission indicate that this seems to occur around the time of birth, with low neonatal morbidity.^8^ Although neonatal SARS‐CoV‐2 infection is rarely of significant severity, there remains concern surrounding disruption of the pregnancy during the 2nd trimester, as was observed during outbreaks of similar coronaviruses SARS and MERS.^9^, ^10^ Additionally, there are concerns regarding the impact of fetal infection during critical developmental phases, observed in other viral infections such as cytomegalovirus. Therefore, understanding the potential for, and impact of, mid‐gestation fetal infection remains an ongoing public health question. Various mechanisms of vertical transmission have been hypothesised: direct infection of syncytiotrophoblast with subsequent transmission through the cytotrophoblast has been demonstrated in several histopathological and immunological studies^11^, ^12^, ^13^ and appears not to involve the fetal‐origin Hofbauer cells in the majority of transplacental infections.^14^ Infection via trafficked maternal cells has been suggested, as evidenced by an observed expression of ACE2 protein in infiltrating maternal cells in human placentas with chorioamnionitis.^15^ However, it is of note that despite widely reported placental pathology in viraemic mothers, infection in the fetus has not been well characterised and does not seem to be a common feature of preterm birth in association with a confirmed infection in the mother.^16^ Neonatal infection plausibly could occur either through an ascending infection through the vagina and cervical canal or during vaginal birth via direct infant contact with maternal virus.^17^ It is worth noting, however, that a number of case series demonstrate negative vaginal swabs in the presence of confirmed maternal SARS‐CoV2 pneumonia.^18^, ^19^

To explore the susceptibility of the mid‐gestation human fetus to SARS‐CoV‐2 infection, we studied multiple fetal tissues at different gestational stages. We initially probed publicly available single‐cell RNA sequencing data from fetal tissues at different developmental stages (10–18 post‐gestational weeks [PCW]). We then validated our findings by exploring gene expression levels of ACE2 and TMPRSS2 via quantitative PCR analysis of the fetal tissues that could be exposed to virus *in utero* and examined expression at protein level using multicolour immunohistochemistry. This enabled us to explore both expression of proteins implicated in SARS‐CoV‐2 infection and their tissue colocalisation. Finally, we studied amniotic membrane and placental samples of matching developmental stages and at term, to identify potential viral entry points at the maternal–fetal interface.

## Methods

### Samples and ethics

Human fetal tissues were obtained with consent through the Human Developmental Biology Resource (HDBR; REC 18/LO/0822 – IRAS 244325; Project ID 200568). Fetal samples were obtained from fetuses ranging from 14 to 22 PCW (12–20 post menstrual weeks of gestation). Fetal staging was calculated based on foot length (mm) and referred to as PCW. The tissue library included fetal lung, intestine, kidney, bladder, skin, amniotic membrane and placenta (CV, chorionic villi). Placental samples were obtained at delivery of an uncomplicated, full‐term pregnancy (median 39 weeks PCW, range 38^+1^ to 39^+4^) for six patients recruited through the EVERREST Prospective Study as normal controls (National Research Ethics reference 13/LO/1254), NCT02097667 registered 31 October 2013.^20^ Tissue staining and RNA expression level was referenced to that of human adult lung controls, commercially obtained from Insight Biotechnology and Takara.

### Single‐cell RNA sequencing

Suitable Single‐Cell RNA sequencing datasets were identified in public repositories from broad high‐impact Single‐Cell studies (available at www.proteinatlas.org and https://descartes.brotmanbaty.org/) ^21^, ^22^. These covered human fetal specimens from multiple gestational ages, as well as human adult reference samples. For the adult data, a normalised count matrix (i.e. TPM normalised) was downloaded, containing samples from different origins, annotated by cell‐type. Fetal data were downloaded as a raw count matrix, with analysis carried out after random sampling of 5000 cells per cell type per organ (in cases where <5000 cells of a given cell type were represented in a given organ, all cells were taken). The sampled data comprise gene count information for 377 456 cells across 15 organs. The data was then CPM‐normalised, and log‐transformed using the Seurat R package (satijalab.org/seurat). A custom R script was then used to extract and plot (using the ggplot2 package by tidyverse, ggplot2.tidyverse.org) the expression data for ACE2 and TMPRSS2 across a range of fetal tissues (adrenal, heart, lung, cerebellum, intestine, muscle, spleen, cerebrum, kidney, pancreas, stomach, eye, liver, thymus and placenta) and time‐points between 10 and 18 PCW.

### Gene expression analysis via RT‐qPCR

Whole tissue gene expression was analysed by Real‐Time quantitative Polymerase Chain Reaction (RT‐qPCR). First, RNA was extracted from formalin‐fixed paraffin‐embedded tissue blocks (RNAEasy FFPE Kit; 73504, Qiagen, Hilden, Germany). The cDNA was then generated upon retro‐transcription following the manufacturer’s recommendation (Super Script VILO IV Master Mix; 11756050, Life Technologies, Carlsbad, CA, USA). RT‐qPCR was performed using an Applied Biosystem Step One v23 machine, utilising SYBR Green (Agilent, 600882, Santa Clara, CA, USA) and predesigned KiCqStart primers for ACE2, TMPRSS2 and GAPDH (Merck, Darmstadt, Germany). Primer sequences are available in Table S1. Relative expression of the analysed genes was calculated based on Δ*C*_T_ values, compared with the adult lung expression level as positive control, using the ∆∆*C*_T_ Livak method.^23^ Statistical significance was assessed by non‐parametric one‐sample Wilcoxon signed rank test against the positive adult lung control.

### Two‐colour immunohistochemistry to assess protein localisation

Tissue slides were analysed by two‐colour immunohistochemistry. Staining was performed utilising primary antibodies for ACE2 (R&D Systems, Minneapolis, Minnesota, USA, MAB933, RRID:AB_2223153; Mouse anti‐Human 1:200 with red chromogen) and TMPRSS2 (Abcam, Cambridge, UK, ab109131, RRID:AB_10863728; rabbit anti‐human 1:500 with Brown chromogen). The Leica refine polymer kit (Leica, Wetzlar, Germany, DS9800, RRID:AB_2891238) and refine red kit (DS9390) were utilised to stain the antibody–antigen complex. Sections were counterstained with haematoxylin to highlight the localisation of the nuclei. Staining specificity and quality were evaluated by the Department of Histopathology of Great Ormond Street Hospital. Qualitative analysis of the staining localisation within each tissue was performed to assess localisation and co‐localisation of the staining. A representative panel showing negative controls and single colour staining is presented in Figure S3. Quantitative analysis was performed using QuPath:^24^ specifically, tissue sections were segmented using a semi‐automated pixel classifier. Positively stained areas for each chromogen were measured using machine‐learning based pixel classifiers and the percentage of staining area relative to total tissue area was computed. Double positive expression was defined by the percentage of staining areas that co‐expressed ACE2 and TMPRSS2. This was then compared between samples across different gestational stages and compared with adult lung sections as positive control reference.

### Statistical analyses

Gestational expression changes were explored using linear regression modelling comparing expression level and developmental stages. We obtained and analysed seven tissues from six fetal samples, in addition to seven term placenta samples. Statistical significance between each tissue and the adult lung expression threshold was assessed, given the internal positive control of adult lung RNA used as a reference for each PCR plate; the pooled mean and standard deviation of all tissue samples from each gestational time point were compared with this value in a one‐sample *t*‐test. The ACE2 and TMPRSS2 levels were graphically depicted as the mean of the fold change in expression in each tissue. The temporal variation for each tissue is also presented as individual graphs, simple linear regression with 95% CI was performed to illustrate temporal trends. Statistical analyses were performed using Microsoft EXCEL (Microsoft Corporation, WA, USA) and GraphPad PRISM v9.0 (GraphPad Software, CA, USA). Data are displayed in the text as Mean ± SEM unless otherwise stated, and a *P*‐value of <0.05 was accepted as denoting statistical significance.

## Results

### Analysis of scRNA‐sequencing data

**Figure 1:**
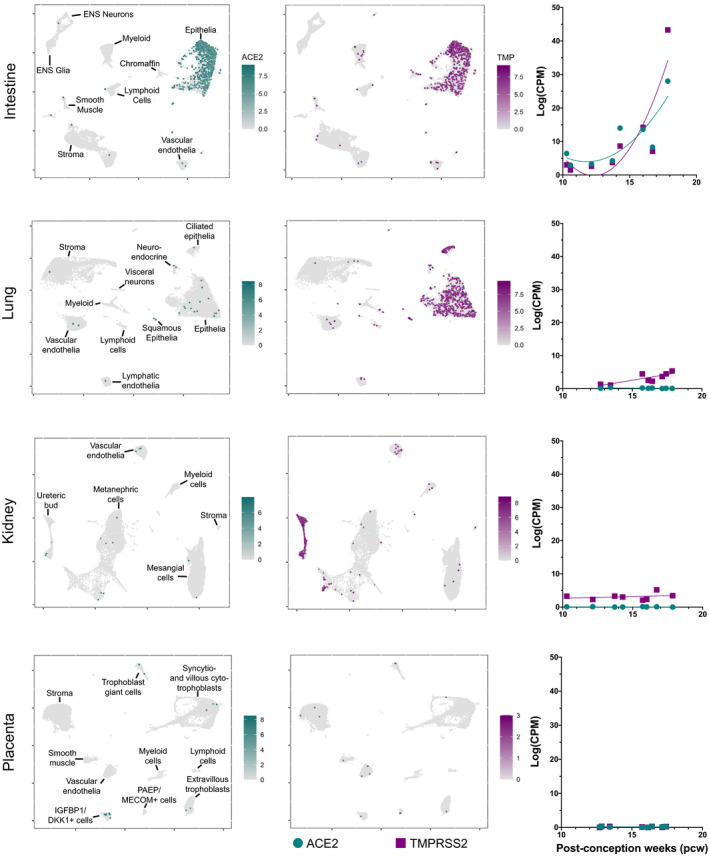
Retrospective analysis performed on public repository single‐cell RNA sequencing data, investigating the expression of ACE2 (Cyan) and TMPRSS2 (Purple). The UMAPs on the left depict the clusters of cells expressing ACE2 and TMPRSS2 in the fetal intestine, lung, kidney and placenta. The plots on the right, show a regression analysis on the variation in the expression levels of the two genes of interest, across the gestational stages analysed.

Our analysis of public repository data identified the human fetal intestine as highly co‐expressing both ACE2 and TMPRSS2. Upon interrogation of expression in the different specific cell types, we identified the intestinal epithelial cells as being the main contributors to overall intestine positivity for these genes (Figure 1). This analysis also indicated that whereas other tissues, notably kidney and lung, showed expression of ACE2, the lack of co‐expression with TMPRSS2 suggests these tissues are unlikely to be susceptible to SARS‐CoV‐2 infection during gestation. Co‐expression of ACE2 and TMPRSS2 in the intestine increased during gestation in an exponential manner across the first and second trimester timepoints studied (10–18 PCW). None of the other tissues analysed showed such significant increase in gene expression over the gestational age range for which data were available. In line with this observation, the adult intestine also showed the greatest co‐expression. This was further supported by analysis of cell‐specific expression, with the greatest expression in adult intestinal enterocytes. Overall, this analysis of open access RNA sequencing atlas data suggests that transcription of ACE2 and TMPRSS2 differs markedly in human fetal subjects compared with what has been reported for children and adults.

### Analysis of gene expression at tissue level across the second trimester

**Figure 2:**
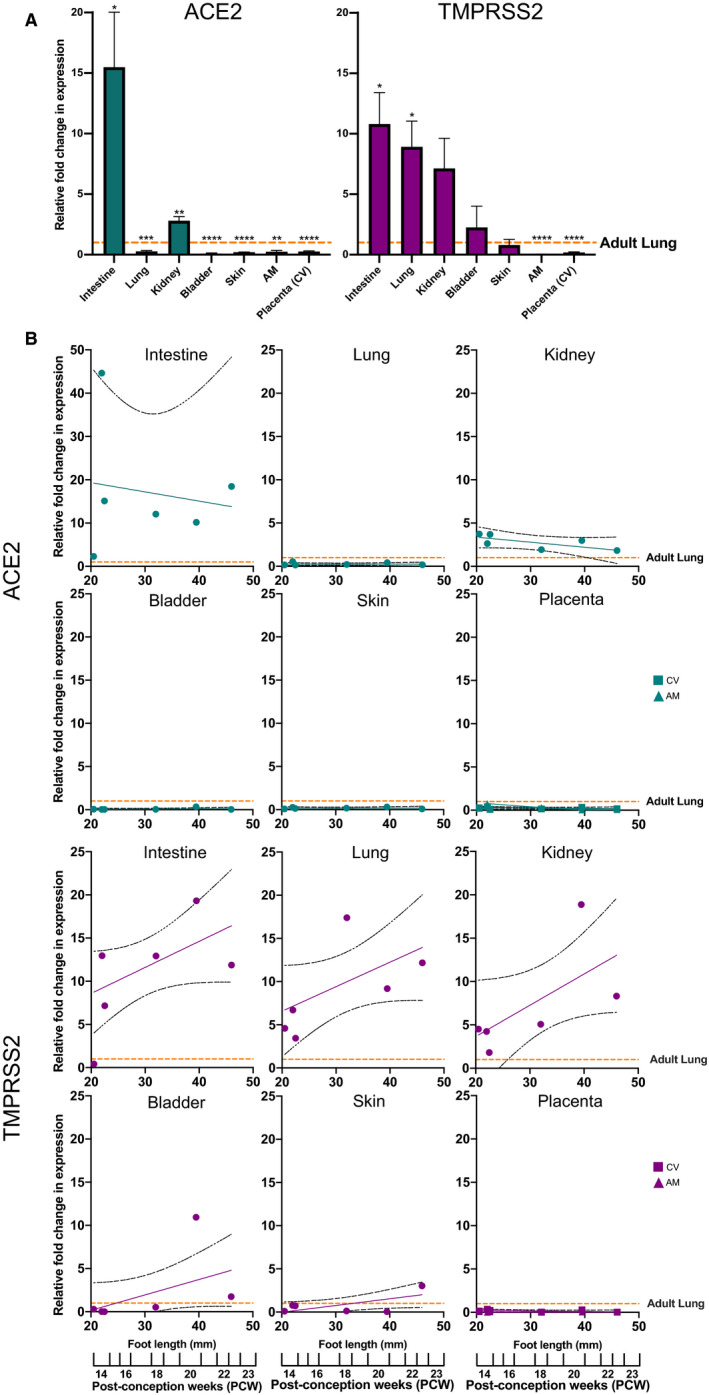
Graphs depicting the results of an RT‐qPCR analysis performed on human fetal and placental tissues (AM, amniotic membrane; CV, chorionic Villi) at different developmental stages. (A) The gene expression of ACE2 and TMPRSS2 is presented as the log‐fold change relative to the adult lung, averaged across the gestational ages studied (*n* = 6 for each tissue). Data are presented as mean ± SEM; *P < 0.05, **P < 0.005, ***P < 0.0005, ****P < 0.0001. (B) Linear regression analysis performed on the expression data shown in (A) to determine variations in expression across the second trimester (14–22 PCW). The data are presented as relative expression to the adult lung.

RT‐qPCR gene expression analysis was carried out on a library of fetal tissues (ranging from 14 to 22 PCW) that are potentially susceptible to SARS‐CoV‐2 infection (Figure 2, Table 1). Using the adult lung as reference, the following was observed. The fetal intestine demonstrated the highest relative expression of ACE2 and TMPRSS2, (fold change relative to adult lung: ACE2 15.49 ± 4.54, *P* = 0.0243 and TMPRSS2 10.80 ± 2.61, *P *= 0.0132). The fetal lung also expressed significant TMPRSS2 expression but showed significantly lower ACE2, far below that observed in the adult lung. Although the fetal kidney showed increased expression of ACE2 and TMPRSS2, the latter did not reach statistical significance. In contrast, the skin and bladder both showed ACE2 expression significantly below that of the adult lung, and TMPRSS2 levels similar to that of the adult lung. Placental tissues showed significantly lower expression than adult lung for both ACE2 and TMPRSS2.

**Table 1:** Summary of the RT‐qPCR data depicted in Figure 2, showing mean, standard deviation (SD), standard error of means (SEM), sample number (*n*), statistical test (one‐sample *t*‐test)

Sample	ACE2						TMPRSS2					
	Mean	SD	SEM	*n*	t‐Test vs Adult Lung	*R* ^2^	Mean	SD	SEM	*n*	t‐Test vs Adult Lung	*R* ^2^
Intestine	15.49	11.13	4.54	6	*P *= 0.0243	0.0240	10.80	6.39	2.61	6	*P *= 0.0132	0.1420
Lung	0.28	0.16	0.07	6	*P *= 0.0001	0.0002	8.92	5.22	2.13	6	*P *= 0.0137	0.2087
Kidney	2.80	0.83	0.34	6	*P *= 0.0031	0.1478	7.14	6.11	2.50	6	*P *= 0.0573	0.2401
Bladder	0.09	0.12	0.05	6	*P *≤ 0.0001	0.0386	2.26	4.31	1.76	6	*P *= 0.5071	0.1895
Skin	0.17	0.09	0.04	6	*P *≤ 0.0001	0.0005	0.80	1.14	0.47	6	*P *= 0.6852	0.2511
Amniotic membrane	0.25	0.27	0.11	6	*P *= 0.0010	0.2268	0.03	0.03	0.01	6	*P *≤ 0.0001	0.0725
Placenta	0.25	0.12	0.05	6	*P *≤ 0.0001	0.0399	0.18	0.14	0.06	6	*P *≤ 0.0001	0.0888
Term placenta	0.45	0.26	0.10	7	*P *= 0.0013		0.28	0.11	0.04	7	*P *≤ 0.0001	

### Analysis of protein expression at tissue level across the second trimester

**Figure 3:**
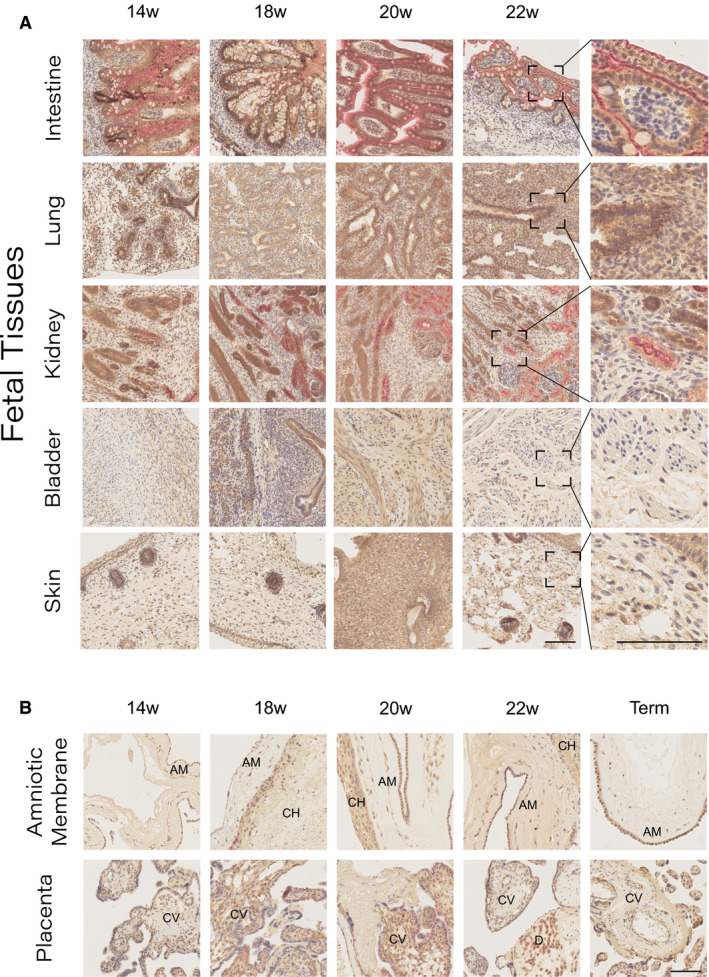
Two‐colour immunohistochemical staining for ACE2 (Red) and TMPRSS2 (Brown). Sections were counterstained with haematoxylin (Purple) to highlight the localisation of the cell nuclei. (A) Antigen localisation in the fetal intestine, lung, kidney, bladder and skin. The last column shows a higher magnification to highlight the staining localisation. Scale bars: 100 µm. (B) Antigen expression in the placental tissues across the second trimester (14–22 PCW), as well as at term. Placental sampling was performed to investigate expression in the amniotic membrane and chorionic villi structures. AM, amnion; CH, chorion; CV, chorionic villi; D: decidua. Scale bar: 100 µm.

Two‐colour immunohistochemistry was used to investigate expression and localisation of ACE2 and TMPRSS2 proteins in multiple fetal tissues across second trimester. Tissues positively expressing the antigens showed localisation of TMPRSS2 (Brown) across the plasma membrane of the epithelial cells, whereas ACE2 (Red) localised specifically to the apical membrane of the epithelia (Figure 3). These data validate the mRNA findings confirming little to no ACE2 expression in fetal skin, bladder and lung, with lung and bladder expressing detectable levels of TMPRSS2. The fetal intestine and kidney specimens showed a strong protein co‐expression, with the intestine having the most significant co‐localisation on the mucosal epithelium, highlighted in the high magnification images (Table 2, Figure 3A, Right and Figure S2). Placental samples expressed low to absent ACE2, whereas TMPRSS2 seemed to be present at variable levels in the chorionic villi; a finding that is consistent across the gestational window of study but which did not match the gene expression data by tissue‐level PCR. The lack of co‐localisation with ACE2 was also true for the term placenta samples (Figure 3B).

**Table 2:** Summary of the image quantification analyses performed using QuPath on the images shown in Figure 3 and highlighting the gestational age (GA), percentage of tissue expressing TMPRSS2 and ACE2, as well as their co‐localisation (%) across the total area analysed (mm^2^)

Sample	GA (PCW)	ACE2 (%)	TMPRSS2 (%)	Co‐localisation (%)	Area (mm^2^)
Intestine	14	9.2	24.4	1.2	53.4
	18	0.2	45.1	0.0	9.4
	20	15.3	25.3	2.8	109.3
	22	8.4	31.9	0.6	10.9
	Average	8.3	31.7	1.2	45.8
Lung	14	0.0	46.9	0.0	4.7
	18	0.0	24.6	0.0	31.6
	20	0.6	48.3	0.0	190.8
	22	0.0	27.0	0.0	49.6
	Average	0.2	36.7	0.0	69.2
Kidney	14	4.0	46.9	0.5	6.0
	18	3.1	38.6	0.4	32.9
	20	4.5	45.7	0.3	81.3
	22	1.9	21.3	0.1	75.3
	Average	3.4	38.1	0.3	48.9
Bladder	14	0.0	2.0	0.0	7.2
	18	0.0	8.5	0.0	8.1
	20	0.0	65.1	0.0	0.5
	22	0.0	2.4	0.0	50.0
	Average	0.0	19.5	0.0	16.5
Skin	14	0.0	12.7	0.0	3.4
	18	0.0	14.0	0.0	10.5
	20	0.0	51.2	0.0	10.4
	22	0.0	37.1	0.0	3.6
	Average	0.0	28.8	0.0	7.0
Amniotic membrane	14	0.0	4.0	0.0	8.9
	18	1.2	3.2	0.0	32.7
	20	0.9	27.6	0.7	85.4
	22	0.0	10.1	0.0	17.3
	38+	0.1	16.2	0.0	10.4
	Average	0.6	14.3	0.2	36.5
Placenta	14	0.0	21.3	0.0	3.3
	18	0.4	13.0	0.1	19.1
	20	1.0	17.7	0.1	126.8
	22	0.5	9.4	0.0	78.9
	38+	0.4	14.7	0.0	37.2
	Average	0.6	13.7	0.1	65.5

## Discussion

In this study, we assess the expression of ACE2 and TMPRSS2 at the gene and protein level, across a library of human fetal tissues obtained from the second trimester (14–22 PCW) to term. Our findings demonstrate that most of the fetal tissues analysed lack expression (or co‐localisation) of the proteins required for SARS‐CoV‐2 infection. However, two tissues (fetal kidney and intestine) manifested co‐expression of both target proteins. As the fetal kidney is not anatomically directly exposed to the amniotic fluid, we believe it is unlikely to be a relevant route for viral infection. Haematogenous spread as a mechanism of infection and transmission across the placenta into the fetal circulation appears unlikely, due to inefficient viral replication in placental tissues.^25^ The fetal intestine, directly exposed to the amniotic fluid via fetal swallowing, shows an increase in the expression of ACE2 throughout gestation with a high level of TMPRSS2 expression in the mucosa. Results from publicly available fetal single cell atlas data support our findings,^22^ allowing extension of these observations to 8 PCW, before the gestational age that fetal swallowing is known to begin. The intestinal findings are also in line with recent demonstration that the human fetal stomach highly co‐expresses proteins required for SARS‐CoV‐2 infection and that fetal stomach‐derived organoids are susceptible to viral infection.^26^ ACE2 expression has also been reported in an immunohistochemistry study of fetal ileum and rectum samples from 15 weeks of gestation,^27^ in which the authors also noted ACE2 expression in the fetal kidney but none in the fetal brain or heart. Taken together, these results suggest that both the upper and lower gastrointestinal tract may be a potential entry route for SARS‐CoV‐2 into the second‐trimester human fetus. This is also consistent with our additional retrospective analysis, performed on all the other fetal tissues present in the reference single cell atlas,^22^ indicating that the co‐expression of the two genes is also present in the stomach (Figure S1).

Previous studies have assimilated data available from expression profiles on scRNA atlas in the public domain to demonstrate ACE2/TMPRSS2 expression in human fetal tissues and at the maternal‐fetal interface.^28^ However, these studies have not explored expression within the fetal intestine, and lack validation at a protein level.^29^ To the best of our knowledge, this is the first work to explore ACE2/TMPRSS2 both at gene expression and protein levels in the human fetus, and specifically in the second trimester of pregnancy.

Our findings support clinical observations from meta‐analyses that the incidence of COVID‐19‐related fetal complications during pregnancy is low given the number of adult COVID‐19 cases worldwide (234 million confirmed cases, 4.8 million deaths at the time of writing: https://github.com/CSSEGISandData/COVID‐19), including large numbers of pregnant women. Comparatively, in previous coronavirus epidemics, far fewer cases have been reported, albeit with a higher frequency of fetal adverse events.^30^ The UK Obstetric Surveillance System reports that pregnant women in later gestation are not protected from severe infection;^31^ with an increased frequency of iatrogenic preterm birth, possibly due to severe maternal illness. There have been speculative data published suggesting that women may be at risk of severe SARS‐CoV2‐related illness in the peripartum period, which has led to regional policy that the non‐compromised fetus should remain undelivered if possible.^32^, ^33^

This study contains novel data that corroborate real world observations, but it nonetheless is important to recognise a few limitations of the data we present. The Human Developmental Biology Resource includes human fetal tissues for research purposes only up to 22 weeks of gestation, therefore we are unable to investigate the expression of ACE2 and TMPRSS2 in fetal tissues past this time point. Furthermore, we have not directly validated the susceptibility to infection in the tissues of interest with infection assays; work with live SARS‐CoV‐2 virus is beyond the scope of this study. Previous collaborative studies from our group have demonstrated infection in tissue and organoids derived from human fetal stomach demonstrating that a high expression at both RNA and protein level corresponds to a significant propensity for viral infection.^26^ Hence, the proposed route of infection via the gastrointestinal tract is reasonable, in keeping with others’ published work on the adult intestine.^34^ Furthermore, this corresponds to proposed mechanisms for spread among younger children, as gastrointestinal symptoms and fecal shedding are more characteristic of infection in younger patients,^35^, ^36^, ^37^ and may possibly be associated with a milder disease phenotype.^38^

The hypothesis of intrauterine infection through the gastrointestinal tract requires viral entry to the fetal gastrointestinal lumen through fetal swallowing of infected amniotic fluid, and presumes that the amniotic fluid can contain infectious viral particles. Our data indicate that term placenta expresses low to absent ACE2, a key protein for SARS‐CoV‐2 infection, making it difficult for the virus to pass from the maternal blood to the fetus or amniotic fluid. In one study of 31 mothers with COVID‐19, there were only two cases of vertical transmission in which the viral genome was detected in term placentas, and these cases were associated with strong maternal pro‐inflammatory response.^39^ In contrast, single cell transcriptomic studies of early placenta (6–14 post menstrual age gestational weeks) reported co‐expression of ACE2 and TMPRSS2 in stromal and perivascular cells in decidua, and villous cytotrophoblast and syncytiotrophoblast.^28^ It is therefore plausible that women infected early in pregnancy could potentially pass SARS‐CoV‐2 to the fetus, but with low fetal infection rates due to the minimal ACE2 and TMPRSS2 co‐expression in the fetal tissues studied, including the gastrointestinal tract, early in pregnancy. Furthermore, placental pathology including fibrin deposition and intervillous thrombosis associated with preterm labour, have been sporadically observed in SARS‐CoV2‐positive mothers, linked to immunochemical evidence of virus expression in the trophoblast.^13^, ^40^ One possible reason for discordant results within the published literature may be the widespread presence of Fc receptors in the human placenta, as these are known to bind antibodies to varying affinities^41^ and may therefore lead to non‐specific staining. This emphasises the need for molecular validation, such as the RT‐qPCR or RNA‐seq data presented here together with the placental immunohistochemistry studies. Regarding perinatal transmission, there have been several studies reporting an absence of detectable virus in genital tract swabs in women with severe symptoms,^18^ or in pregnant women with mild symptoms.^42^, ^43^ However, given the immunological changes occurring around the time of labour,^44^ we would suggest that there is currently inadequate evidence to rule this out as the potential route for infection of neonates who are known to have been infected.^3^, ^4^, ^5^ In conclusion, we would propose that maternal viraemia‐associated presence of virus within the amniotic fluid and birth canal, as reported,^28^, ^33^ may produce an environment where the fetus is susceptible to infection through the gastrointestinal tract, and that this susceptibility may be present from the late second trimester.

### Contribution to authorship

MFMG and PDC conceived the study and designed the experiments with the help of ALD. MAB and JRD contributed equally to this work, conducted the experiments and analysed the data with the help and support of MFMG. FP and DC helped MAB in performing the retrospective sequencing analyses. SS, DS, BCJ, OO and NJS helped with staining optimisation, interpretation and imaging. KM procured the term placental RNA. MAB, JRD, MFMG and PDC wrote this manuscript. All authors contributed to manuscript revision and approved the final version.

### Disclosure of interests

The authors declare no conflict of interests related to this work or its developments. DC is founder, shareholder and consultant of Next Generation Diagnostic SRL.

### Funding

This work was made possible by an MRC/UKRI COVID‐19 Rapid response initiative grant (MR/V028480/1).

## Supporting information


**Figure S1**. A retrospective analysis performed on public repository single‐cell RNA sequencing data, investigating the expression of ACE2 (Cyan) and TMPRSS2 (Purple).

**Figure S2**. Higher magnification panel of the two‐colour immunohistochemical staining for ACE2 (Red) and TMPRSS2 (Brown) presented in Figure 3.

**Figure S3**. Single‐colour immunohistochemistry on tissues obtained from a 14 PCW fetus, and used as control for ACE2 (Red) and TMPRSS2 (Brown) staining and to establish the thresholds for image quantification.

**Table S1**. List of the primers utilised in the RT‐qPCR experiments.

Supplementary Material

Supplementary Material

Supplementary Material

Supplementary Material

Supplementary Material

Supplementary Material

Supplementary Material

Supplementary Material

Supplementary Material

Supplementary Material

Supplementary Material

## Data Availability

Sequencing data derived from public domain resources.
