# Supplementary figures and images for: COVID‐19 and vertical transmission: assessing the expression of ACE2/TMPRSS2 in the human fetus and placenta to assess the risk of SARS‐CoV‐2 infection
Source: BJOG. 2021 Nov 18;129(2):256–66. doi: 10.1111/1471-0528.16974 (PMC8652560; doi:10.1111/1471-0528.16974)

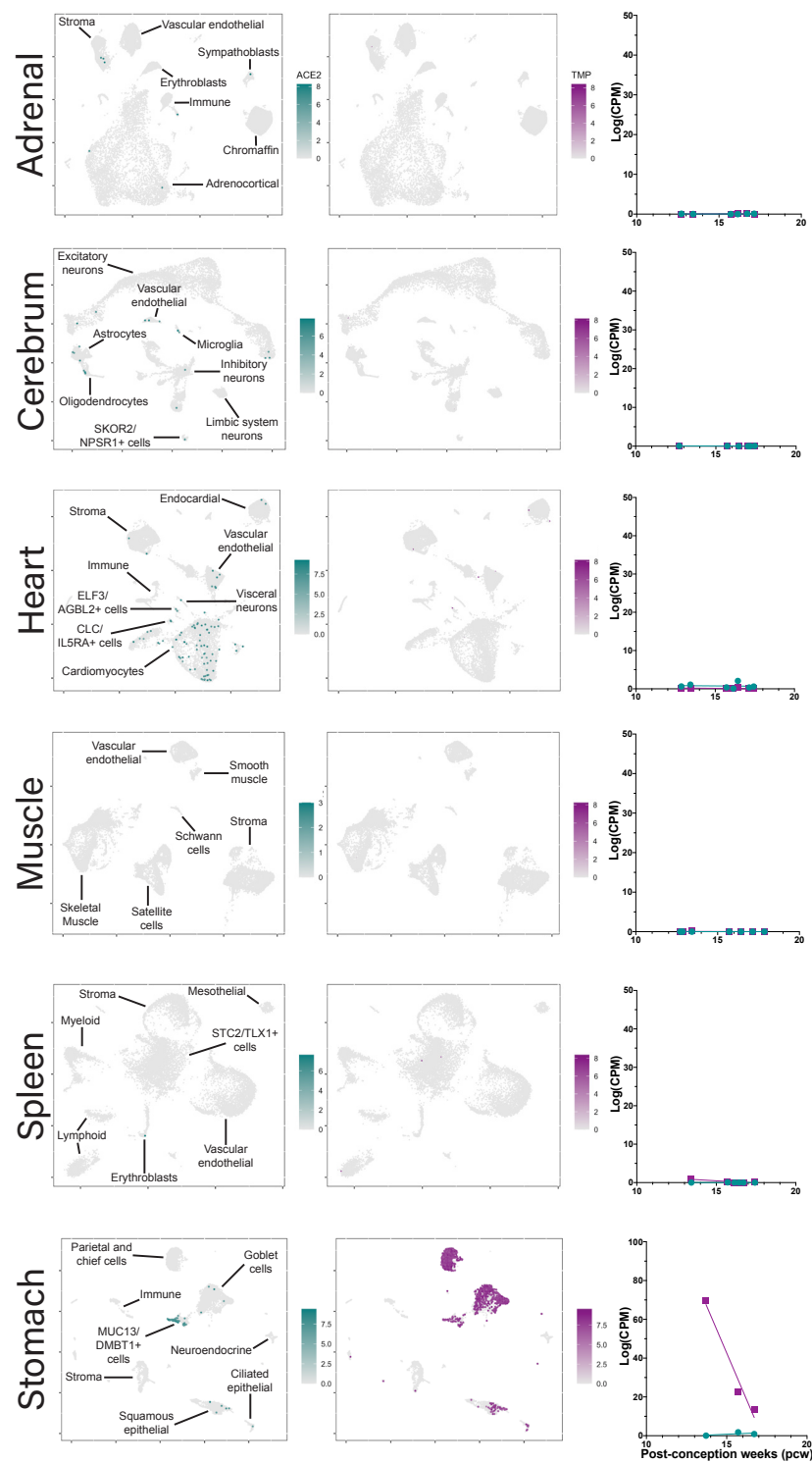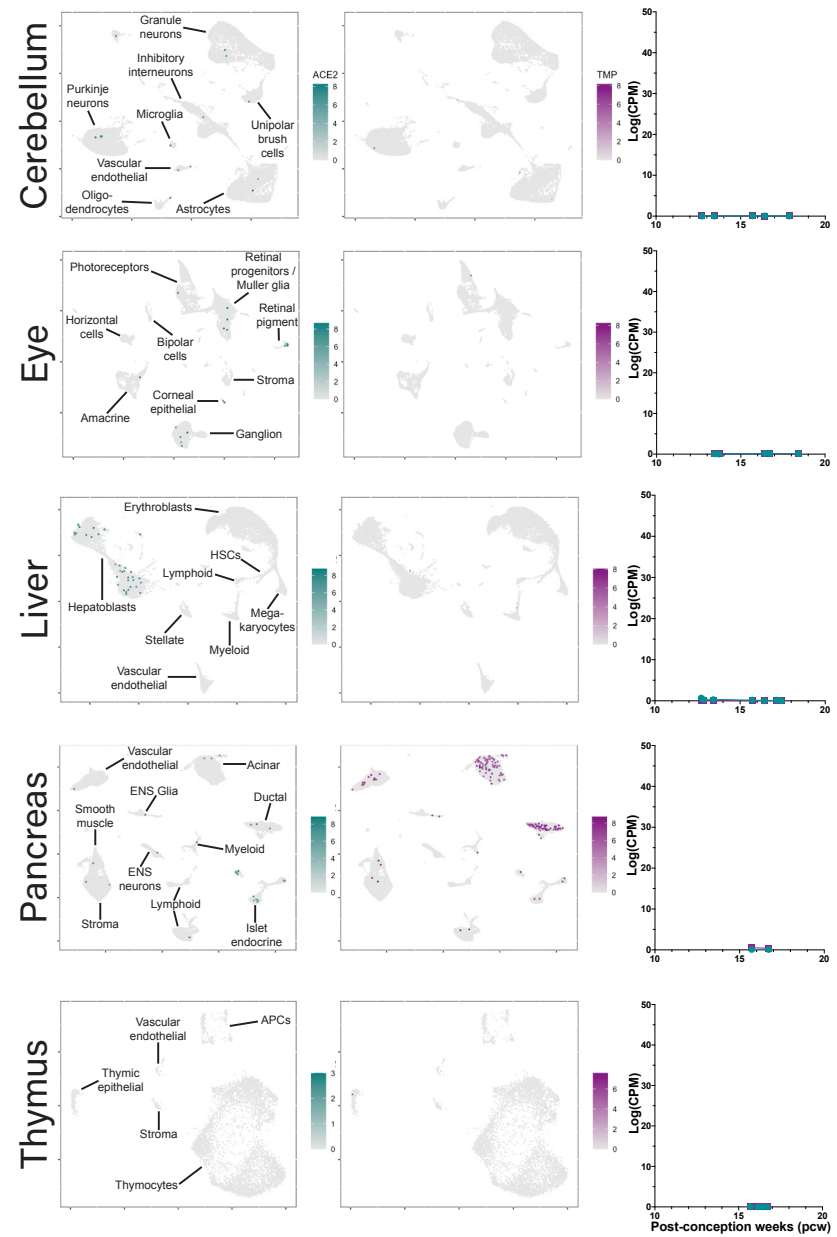

Supplement: Supplementary file 1 — Figure S1. A retrospective analysis performed on public repository single‐cell RNA sequencing data, investigating the expression of ACE2 (Cyan) and TMPRSS2 (Purple). [file BJO-129-256-s011.pdf]

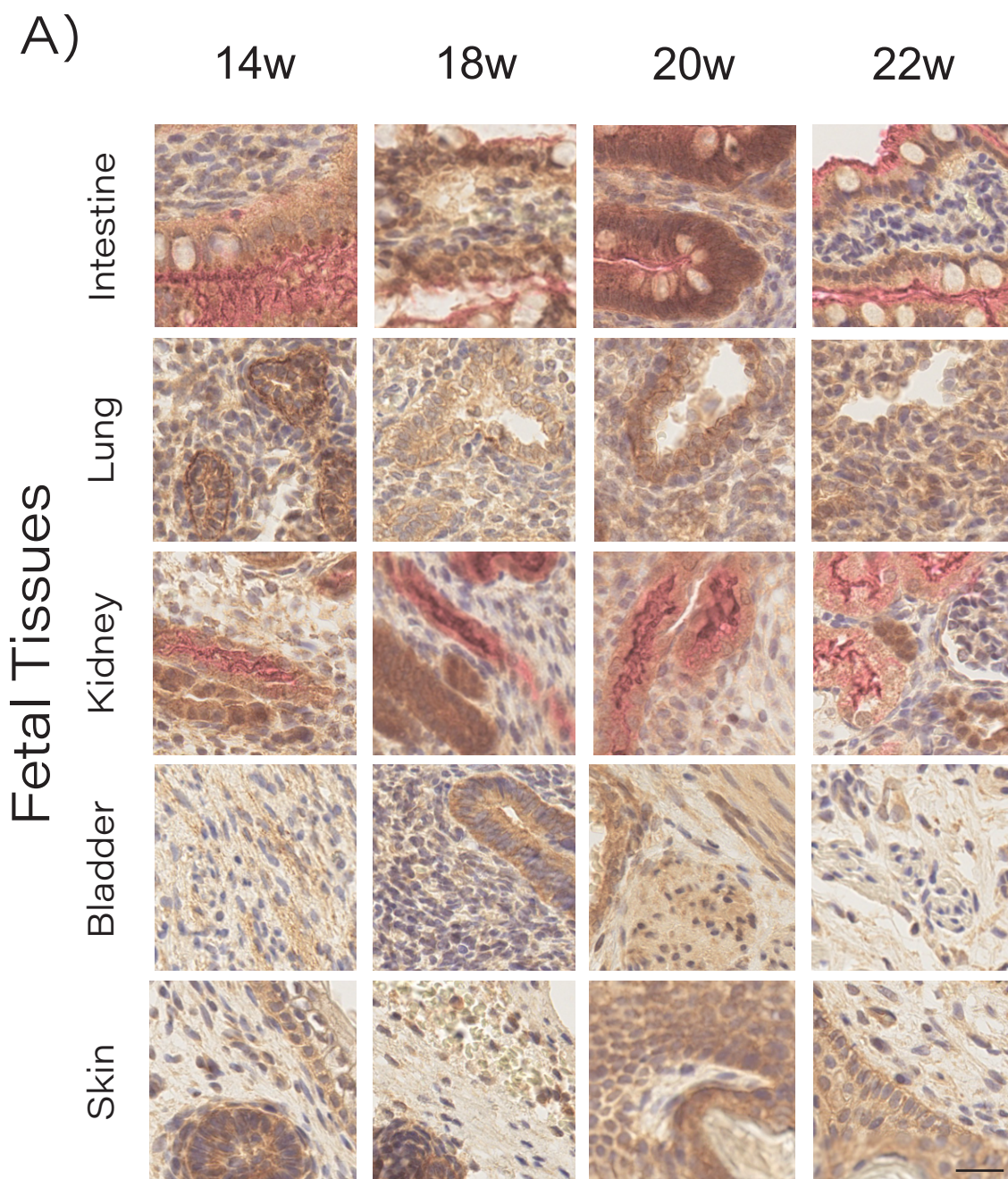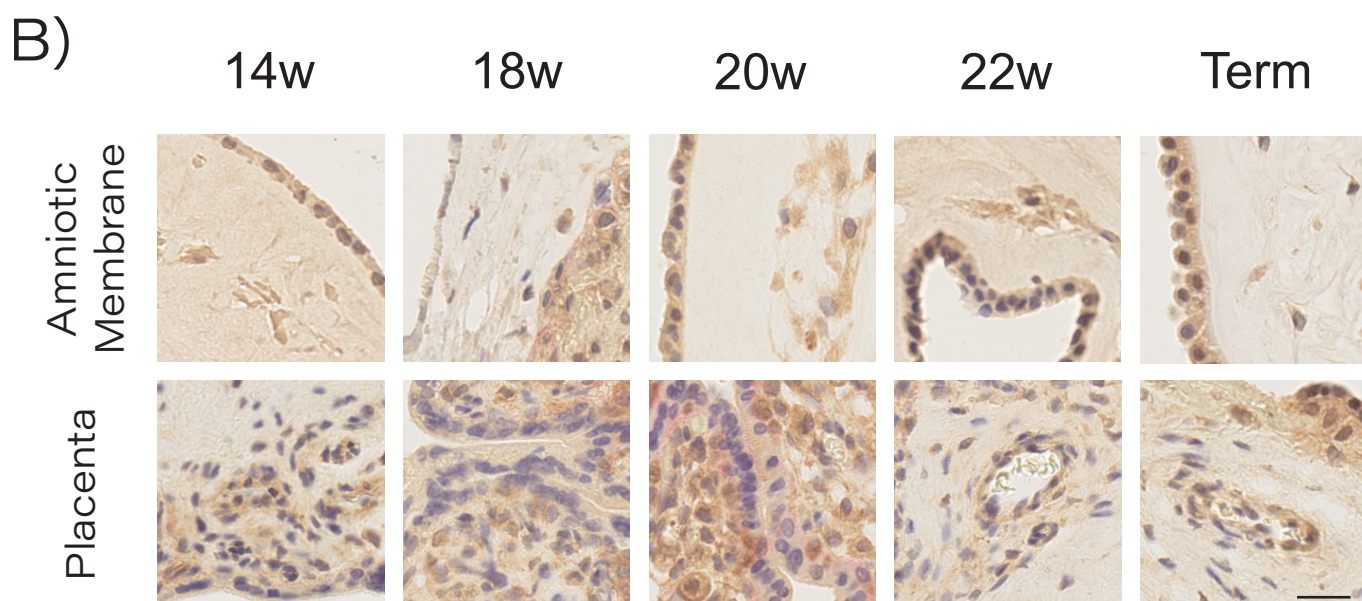

Supplement: Supplementary file 2 — Figure S2. Higher magnification panel of the two‐colour immunohistochemical staining for ACE2 (Red) and TMPRSS2 (Brown) presented in Figure 3. [file BJO-129-256-s001.pdf]

A)

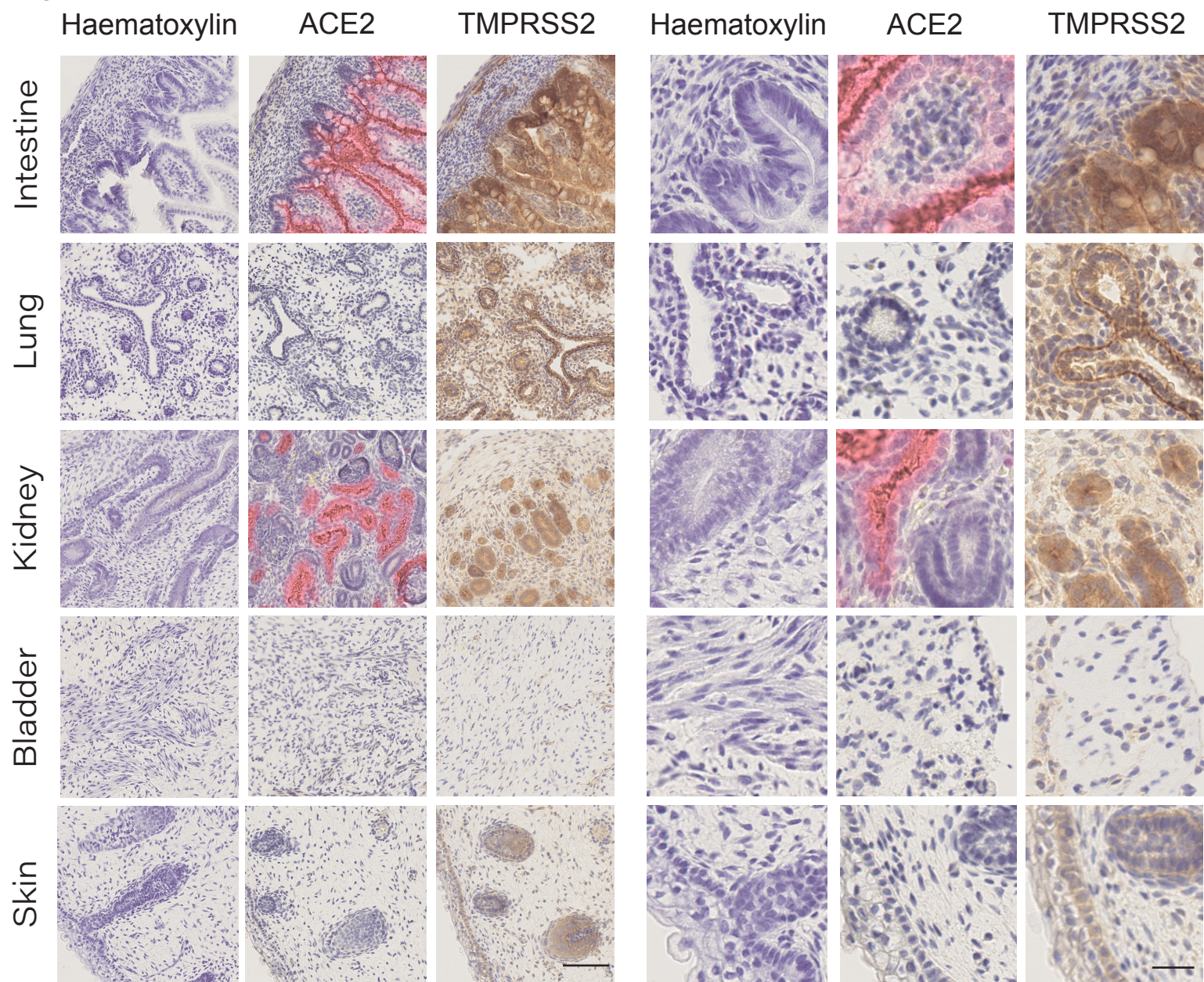

B)

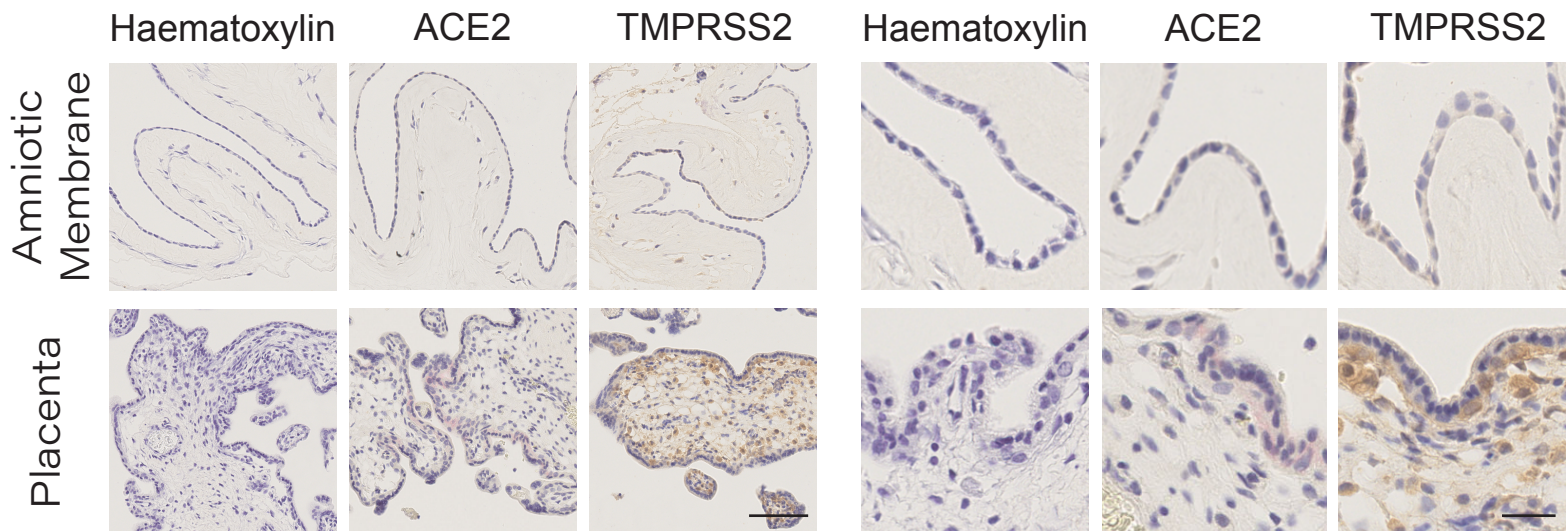

Supplement: Supplementary file 3 — Figure S3. Single‐colour immunohistochemistry on tissues obtained from a 14 PCW fetus, and used as control for ACE2 (Red) and TMPRSS2 (Brown) staining and to establish the thresholds for image quantification. [file BJO-129-256-s014.pdf]
