# Supplementary material for: COVID‐19 and vertical transmission: assessing the expression of ACE2/TMPRSS2 in the human fetus and placenta to assess the risk of SARS‐CoV‐2 infection
Source: BJOG. 2021 Nov 18;129(2):256–66. doi: 10.1111/1471-0528.16974 (PMC8652560; doi:10.1111/1471-0528.16974)
Supplement: Supplementary file 4 — Table S1. List of the primers utilised in the RT‐qPCR experiments. [file BJO-129-256-s013.docx]

| **Target Gene** | **Direction** | **Sequence** |
| --- | --- | --- |
| ACE2 | Forward | 5’- GTTTGTAACCCAGATAATCCAC |
|  | Reverse | 5’- AATGATTTGCTCTTGCCATC |
| TMPRSS2 | Forward | 5’- CAGGTCATATTGAACATTCCAG |
|  | Reverse | 5’ - CTGAGTTCAAAGCCATCTTG |
| GAPDH | Forward | 5’ - TTGATGGCAACAATATCCAC |
|  | Reverse | 5’ - CTTTTGCGTCGCCAG |
