# Supplementary material for: COVID‐19 and vertical transmission: assessing the expression of ACE2/TMPRSS2 in the human fetus and placenta to assess the risk of SARS‐CoV‐2 infection
Source: BJOG. 2021 Nov 18;129(2):256–66. doi: 10.1111/1471-0528.16974 (PMC8652560; doi:10.1111/1471-0528.16974)
Supplement: Supplementary file 5 — Supplementary Material [file BJO-129-256-s010.docx]

| ICMJE DISCLOSURE FORM | |
| --- | --- |
| **Date:** | 5/10/2021 |
| **Your Name:** | Anna David |
| **Manuscript Title:** | COVID-19 and vertical transmission: assessing the expression of ACE2 / TMPRSS2 in the human fetus and placenta to assess the risk of SARS-CoV-2 infection |
| **Manuscript Number (if known):** | **BJOG-21-1162.R1** |
| In the interest of transparency, we ask you to disclose all relationships/activities/interests listed below that are related to the content of your manuscript. “Related” means any relation with for-profit or not-for-profit third parties whose interests may be affected by the content of the manuscript. Disclosure represents a commitment to transparency and does not necessarily indicate a bias. If you are in doubt about whether to list a relationship/activity/interest, it is preferable that you do so.  The author’s relationships/activities/interests should be defined broadly. For example, if your manuscript pertains to the epidemiology of hypertension, you should declare all relationships with manufacturers of antihypertensive medication, even if that medication is not mentioned in the manuscript.  In item #1 below, report all support for the work reported in this manuscript without time limit. For all other items, the time frame for disclosure is the past 36 months. | |

|  | | | **Name all entities with whom you have this relationship or indicate none (add rows as needed)** | **Specifications/Comments (e.g., if payments were made to you or to your institution)** |
| --- | --- | --- | --- | --- |
| **Time frame: Since the initial planning of the work** | | | | |
| **1** | All support for the present manuscript (e.g., funding, provision of study materials, medical writing, article processing charges, etc.)  **No time limit for this item.** | | \|  \| **None** \| \| --- \| --- \|  \| UKRI COVID-19 Rapid response initiative MR/V028480/1 \| £25,000 paid to UCL to do the work in this manuscript \| \| --- \| --- \| \|  \|  \| \|  \| Click the tab key to add additional rows. \| | |
| **Time frame: past 36 months** | | | | |
| **2** | | Grants or contracts from any entity (if not indicated in item #1 above). | \|  \| **None** \| \| --- \| --- \|  \| Innovative Engineering for Health award by the Wellcome Trust (WT101957) “GIFT-Surg” \| *£9,982,215 (to UCL between the EPSRC below and WT). This part supported the salary of one of the co-authors.* \| \| --- \| --- \| \| Engineering and Physical Sciences Research Council (EPSRC)(NS/A000027/1) “GIFT-Surg” \| EPSRC funds to UCL for this jointly funded GIFT-Surg project \| \| European Union Seventh Framework Programme (FP7/2007–2013) grant agreement no. 305823 \| £4,969,421 to UCL to support setting up the ethically approved study to collect placental samples \| | |
| **3** | | Royalties or licenses | \|  \| **None** \| \| --- \| --- \|  \|  \|  \| \| --- \| --- \| \|  \|  \| \|  \|  \| | |
| **4** | | Consulting fees | \|  \| **None** \| \| --- \| --- \|  \| Consultant for Esperare, Geneva, Switzerland for advice on a prenatal drug to treat an inherited skin disorder \| Personal consulting fees \| \| --- \| --- \| \|  \|  \| \|  \|  \| \|  \|  \| | |
| **5** | | Payment or honoraria for lectures, presentations, speakers bureaus, manuscript writing or educational events | \|  \| **None** \| \| --- \| --- \|  \|  \|  \| \| --- \| --- \| \|  \|  \| \|  \|  \| | |
| **6** | | Payment for expert testimony | \|  \| **None** \| \| --- \| --- \|  \|  \|  \| \| --- \| --- \| \|  \|  \| \|  \|  \| | |
| **7** | | Support for attending meetings and/or travel | \|  \| **None** \| \| --- \| --- \|  \|  \|  \| \| --- \| --- \| \|  \|  \| \|  \|  \| | |
| **8** | | Patents planned, issued or pending | \|  \| **None** \| \| --- \| --- \|  \|  \|  \| \| --- \| --- \| \|  \|  \| \|  \|  \| | |
| **9** | | Participation on a Data Safety Monitoring Board or Advisory Board | \|  \| **None** \| \| --- \| --- \|  \|  \|  \| \| --- \| --- \| \|  \|  \| \|  \|  \| | |
| **10** | | Leadership or fiduciary role in other board, society, committee or advocacy group, paid or unpaid | \|  \| **None** \| \| --- \| --- \|  \| Scientific Trustee of Tommy’s charity \| Tommy’s did not fund this work. \| \| --- \| --- \| \| Trustee of the EGA Hospital charity \| EGA Hospital charity did not fund this work \| \| Trustee of Child Health Research Charitable Incorporated Organisation (CIO) \| CIO did not fund this work \| | |
| **11** | | Stock or stock options | \|  \| **None** \| \| --- \| --- \|  \|  \|  \| \| --- \| --- \| \|  \|  \| \|  \|  \| | |
| **12** | | Receipt of equipment, materials, drugs, medical writing, gifts or other services | \|  \| **None** \| \| --- \| --- \|  \|  \|  \| \| --- \| --- \| \|  \|  \| \|  \|  \| | |
| **13** | | Other financial or non-financial interests | \|  \| **None** \| \| --- \| --- \|  \|  \|  \| \| --- \| --- \| \|  \|  \| \|  \|  \| | |
|  | |  |  | |
| **Please place an “X” next to the following statement to indicate your agreement:** | | | | |
|  | | I certify that I have answered every question and have not altered the wording of any of the questions on this form. | | |
